# Clinical and hemodynamic outcomes of side-to-side anastomosis in superficial temporal artery-middle cerebral artery bypass for adult patients with moyamoya disease: a prospective cohort study

**DOI:** 10.3389/fneur.2025.1632626

**Published:** 2025-08-15

**Authors:** Zhiyong Shi, Zhongren Deng, Xinhua Chen, Lingyun Wu, Wei Li, Juan Wang, Yi Liu, Dong Zhang, Yi Wang, Chunhua Hang, Yongbo Yang

**Affiliations:** ^1^Department of Neurosurgery, Nanjing Drum Tower Hospital, Nanjing University Medical School, Nanjing, China; ^2^Department of Neurosurgery, Huai’an Hongze District People’s Hospital, Huai’an, China; ^3^Department of Neurosurgery, West China Hospital, Sichuan University, Chengdu, China; ^4^Department of Neurosurgery, Beijing Hospital, Beijing, China

**Keywords:** moyamoya disease, STA-MCA bypass, S-S bypass, cerebral revascularization, flow hemodynamics, ultrasonography, cerebral ischemia

## Abstract

**Objective:**

Superficial temporal artery-middle cerebral artery (STA-MCA) bypass, characterized by side-to-side (S-S) anastomosis, has been beneficial in reducing the incidence of postoperative complications and recurrent stroke in patients with moyamoya disease (MMD). However, the safety and efficacy of this unconventional S-S procedure remain unclear. This research aimed to investigate the clinical and hemodynamic outcomes associated with the S-S technique.

**Methods:**

Clinical and radiographic data were collected from 50 adult patients with MMD (50 hemispheres), including 23 cases treated with S-S anastomosis and 27 cases treated with end-to-side (E-S) STA-MCA bypass. The patients’ demographic information, clinical presentation, associated medical conditions, intraoperative hemodynamics, postoperative hemispheric perfusion status, and clinical course were obtained through a review of medical records, intraoperative microvascular Doppler ultrasonography (MDU), and postoperative CT perfusion (CTP) imaging.

**Results:**

There was no significant difference between the S-S and E-S groups in baseline characteristics, postoperative complications, bypass patency rate, neovascularization, and modified Rankin Scale (mRS) scores (*p* > 0.05). However, significant differences were noted in bypass time and anastomosis size between the E-S and S-S groups (*p* < 0.001). Intraoperative MDU analysis demonstrated that the mean velocity value (MVV) of the recipient artery entering the Sylvian fissure (RA.ES) and the MVV fold change in donor vessels were significantly higher in the S-S group compared to the E-S group (*p* < 0.05). Postoperative CTP analysis showed no difference in the volume of the infarct core, hypoperfusion, and penumbra between the groups (*p* > 0.05).

**Conclusion:**

The S-S technique demonstrated a different intraoperative self-flow regulation capacity compared to the traditional E-S technique, but it showed no superiority in postoperative hemispheric perfusion and clinical outcomes. The choice of bypass procedure should be individualized.

## 1 Introduction

Extracranial-intracranial (EC-IC) bypass, such as superficial temporal artery-middle cerebral artery (STA-MCA) bypass, characterized by end-to-side (E-S) anastomosis, has been recognized as an effective treatment for moyamoya disease (MMD) (1, 2). The regulation of blood flow and the achievement of an optimal distribution ratio are clinically crucial in the treatment of MMD. According to previous reports, STA-MCA bypass with side-to-side (S-S) anastomosis may reduce the incidence of cerebral hyper-perfusion syndrome (CHS), resulting in mild discomfort symptoms and a short duration (3, 4). However, the safety and efficacy of this novel S-S procedure remain unclear.

At our center, various direct bypass options are available, including E-S anastomosis and S-S anastomosis, with their clinical outcomes and hemodynamic details systematically recorded. In this study, we evaluated the intraoperative hemodynamics, incidence of newly developed complications, changes in hemispheric perfusion, and short-term follow-up outcomes of E-S versus S-S bypass in patients with MMD to identify similarities and differences between the two techniques. We described the unique hemodynamic characteristics observed during the S-S bypass, specifically the “flow shunting” and “flow augmentation” effects, providing a novel perspective for optimizing the application of this surgical technique. To the best of the authors’ knowledge, this is an important contribution that supplements previous research and aids in advancing the understanding of the S-S technique (4).

## 2 Methods

### 2.1 Patient selection

**Figure 1:**
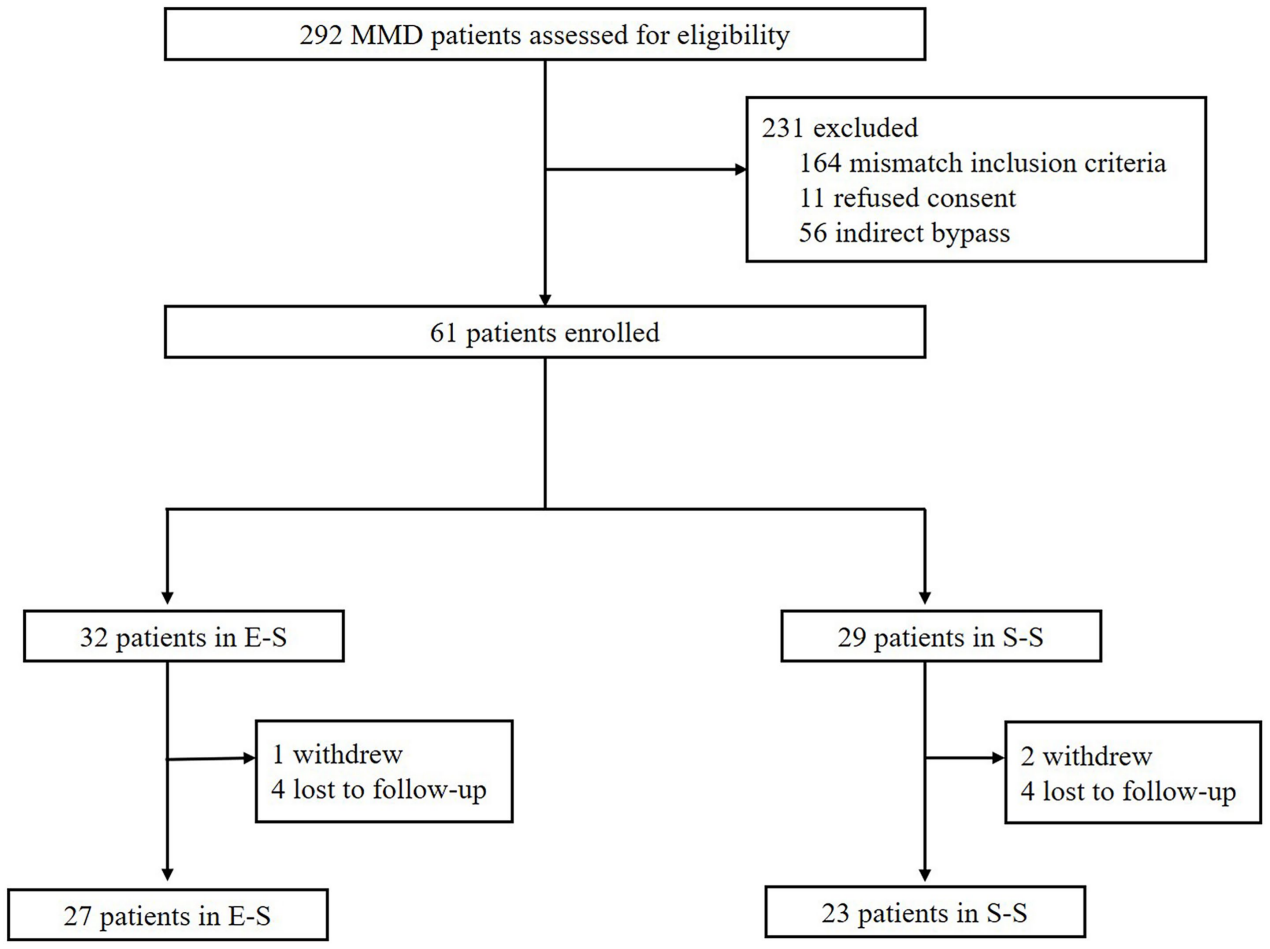
Inclusion and exclusion criteria of the study. E-S, end-to-side; S-S, side-to-side; MMD, moyamoya disease.

This study was approved by the Ethics Committee of Nanjing Drum Tower Hospital (ID: 2022-172-03), and written informed consent was obtained from all participants. The inclusion criteria for the study were as follows (Figure 1): (1) Patients met the guidelines for MMD as proposed by the Research Committee on Spontaneous Occlusion of the Circle of Willis of the Ministry of Health, Labor, and Welfare, Japan (5); (2) The ipsilateral hemisphere demonstrated a time to maximum (*T*_max_) >6 s on preoperative CT perfusion (CTP) imaging; (3) Patients underwent either E-S or S-S STA-MCA bypass; (4) The operated hemisphere was evaluated using intraoperative microvascular Doppler ultrasonography (MDU) and FLOW 800 indocyanine green (ICG) imaging; (5) CTP was conducted at discharge, and digital subtraction angiography (DSA) was performed 6 months postoperatively; and (6) Patients were required to be over 18 years of age. Patients who did not meet these criteria were excluded. A total of 50 hemispheres (50 patients) operated on by a single neurosurgeon (YY) at our center between January 2023 and October 2023 were recruited for this study. Data on patient demographics, radiological imaging, intraoperative hemodynamic details, the incidence of newly developed complications, and clinical follow-up outcomes were recorded and compared.

### 2.2 Data collection and analysis

#### 2.2.1 Patient demographics

The collected patient information included sex, age, onset symptoms, history of hypertension, diabetes, smoking, thyroid function, modified Rankin Scale (mRS) score, presence of intracranial aneurysms, Suzuki stage, presence of moyamoya vessels, operated hemisphere, and bypass time.

### 2.3 Radiological images

#### 2.3.1 CTP images

##### 2.3.1.1 Imaging protocol and analysis

CTP examinations were performed on all enrolled MMD patients using a GE 256-row CT scanner. The scanner settings were 80 kV of voltage and 150 mA, resulting in a slice thickness of 0.625 mm and a matrix size of 256 × 256. Intravenous iodine contrast (5 mL/s, 60 mL total) was administered. Two independent neuroradiologists, blinded to each other’s assessments, analyzed the CTP results using Neuro CT AW4.7 software (Siemens Medical System Workstation) (6). The parameters recorded included cerebral blood flow (CBF), cerebral blood volume (CBV), mean transit time (MTT), and *T*_max_. The cerebellum served as a reference for calculating relative values, focusing on *T*_max_, rCBV, and rCBF as main indices.

##### 2.3.1.2 Data processing

**Figure 2:**
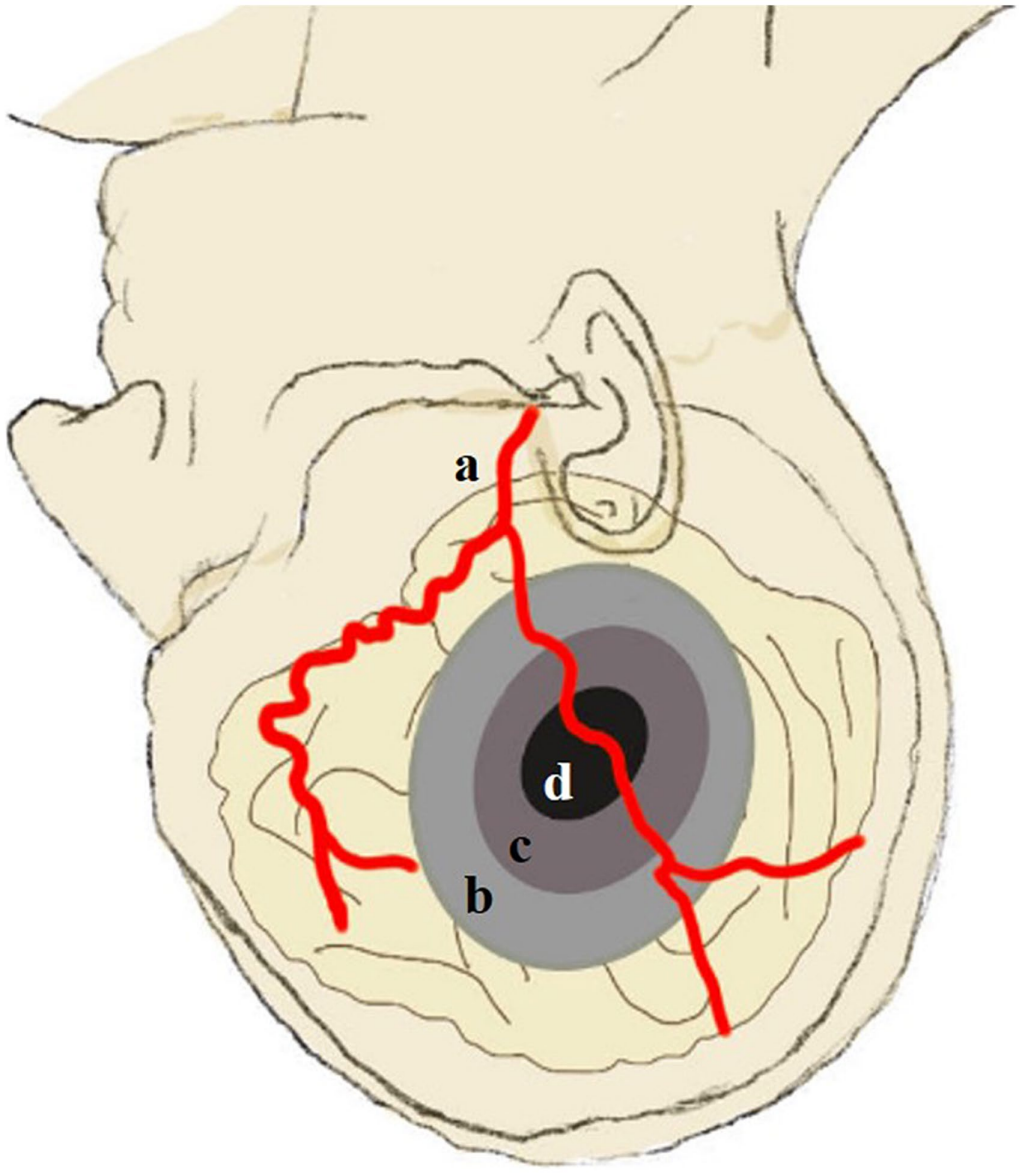
Schematic illustration of hypoperfusion, severe hypoperfusion, infarct core, and penumbra based on CT perfusion scans. (a) The STA. (b) Hypoperfusion area (*T*_max_ >6 s). (c) Severe hypoperfusion area (*T*_max_ >10 s). (d) Infarct core area (rCBF <30%). Hypoperfusion index: (c/b). Mismatch ratio: (b/d). Penumbra: (b–d). CVD: CBV (c/b). STA, superficial temporal artery; CVD, collateral vessel density, CBV, cerebral blood volume.

The infarct core was defined as an rCBF value <30%, and hypoperfusion was defined as a *T*_max_ >6 s. The penumbra was calculated as the volume difference between the hypoperfusion zone and the infarct core. The mismatch ratio was calculated as hypoperfusion zone volume/infarct core volume. Severe hypoperfusion was defined as a *T*_max_ >10 s. The hypoperfusion index was defined as volume (*T*_max_ >10 s)/volume (*T*_max_ >6 s). Collateral vessel density was defined as CBV index (*T*_max_ >10 s)/CBV index (*T*_max_ >6 s) (7). For each case, we defined the volume change percentage of the infarct core, hypoperfusion, and penumbra as [volume_post_ − volume_pre_]/volume_pre_ * 100%. The index of rCBF <30%, *T*_max_ >6 s, *T*_max_ >10 s, *T*_max_ >10 s/*T*_max_ >6 s, penumbra volume, collateral vessel density, and mismatch ratio were compared between the E-S and S-S groups preoperatively and at discharge (Figure 2).

#### 2.3.2 DSA imaging

The evaluation of moyamoya vessels and the Suzuki stage was performed according to previous reports (8). The Suzuki stage was divided into three grades: (1) early stage, which includes hemispheres classified as Suzuki stages I and II; (2) mild stage, which encompasses hemispheres at Suzuki stages III and IV; and (3) advanced stage, comprising hemispheres classified as Suzuki stages V and VI. Moyamoya vessels were divided into three subtypes: (1) none, no obvious moyamoya vessels; (2) sparse, formation of moyamoya vessels at the base of the brain but are limited in distribution; and (3) dense, numerous moyamoya vessels anastomosed to form a network at the end of the ICA and expanded in all directions at the base of the brain. Neo-angiogenesis was graded according to Matsushima as follows: Grade A (>2/3 MCA territory), Grade B (1/3–2/3), and Grade C (<1/3) (9). In addition, the bypass patency rate was also recorded.

#### 2.3.3 Microvascular Doppler ultrasonography examination

##### 2.3.3.1 Surgical procedure

The selection of the bypass technique was primarily determined based on the diameter and bifurcation of the STA, as well as the experience of the neurosurgeon. For STA bifurcations located less than 2 cm from the zygomatic arch, the S-S bypass was preferred, while bifurcations located 2–4 cm from the zygomatic arch can be treated with either the S-S bypass or the E-S bypass. When the bifurcation is more than 4 cm away, the E-S bypass is typically used. In addition, an STA diameter of less than 1 mm favored an E-S bypass, whereas diameters exceeding 1 mm were suitable for either an E-S or an S-S bypass. Moreover, the S-S bypass was preferred in cases where the STA diameter exceeded 2 mm.

MDU examinations were conducted using a standard MDU device (DWL MultiDop X, 16M, Singen, Germany) with a handheld transducer in pulsed-wave mode, all performed in a single laboratory. All examiners followed the same protocol. The patient was operated on under general anesthesia, and a craniotomy was performed to ensure exposure of the recipient artery (RA) in the frontal, temporal, and parietal lobes.

The STA was typically selected as the donor artery (DA), with its parietal branch being the preferred choice. The segment of the DA intended for anastomosis was then dissected and positioned adjacent to the selected RA in either an “S-S” or “E-S” configuration, in preparation for the arteriotomy. The arachnoid membrane over the target RA was incised to a length of approximately 15 mm, and the branching vessels of the RA were meticulously coagulated. The donor artery was placed alongside the RA without tension, ensuring a tension-free state of the vessels. Temporary aneurysm clips were applied to occlude blood flow in both the STA and RA. A micro-scissor was used to make an incision along the longitudinal axis of the vessels. The size of the anastomosis was assessed based on the ratio of the longitudinal axis to transverse axis of the RA incision. A 10-0 nylon suture was employed for the anastomosis. Initially, the two heels of the donor vessel incision were sutured to the corresponding heels of the RA incision, followed by continuous suturing to approximate the two walls. Due to differences in the anastomosis size between E-S and S-S configurations, the E-S group typically required 4 to 5 sutures per side of the vessel wall, whereas the S-S group necessitated 8 to 10 sutures per side. Bypass time was recorded, and patency was confirmed intraoperatively using Flow 800 ICG angiography.

### 2.4 Data collection and analysis

**Figure 3:**
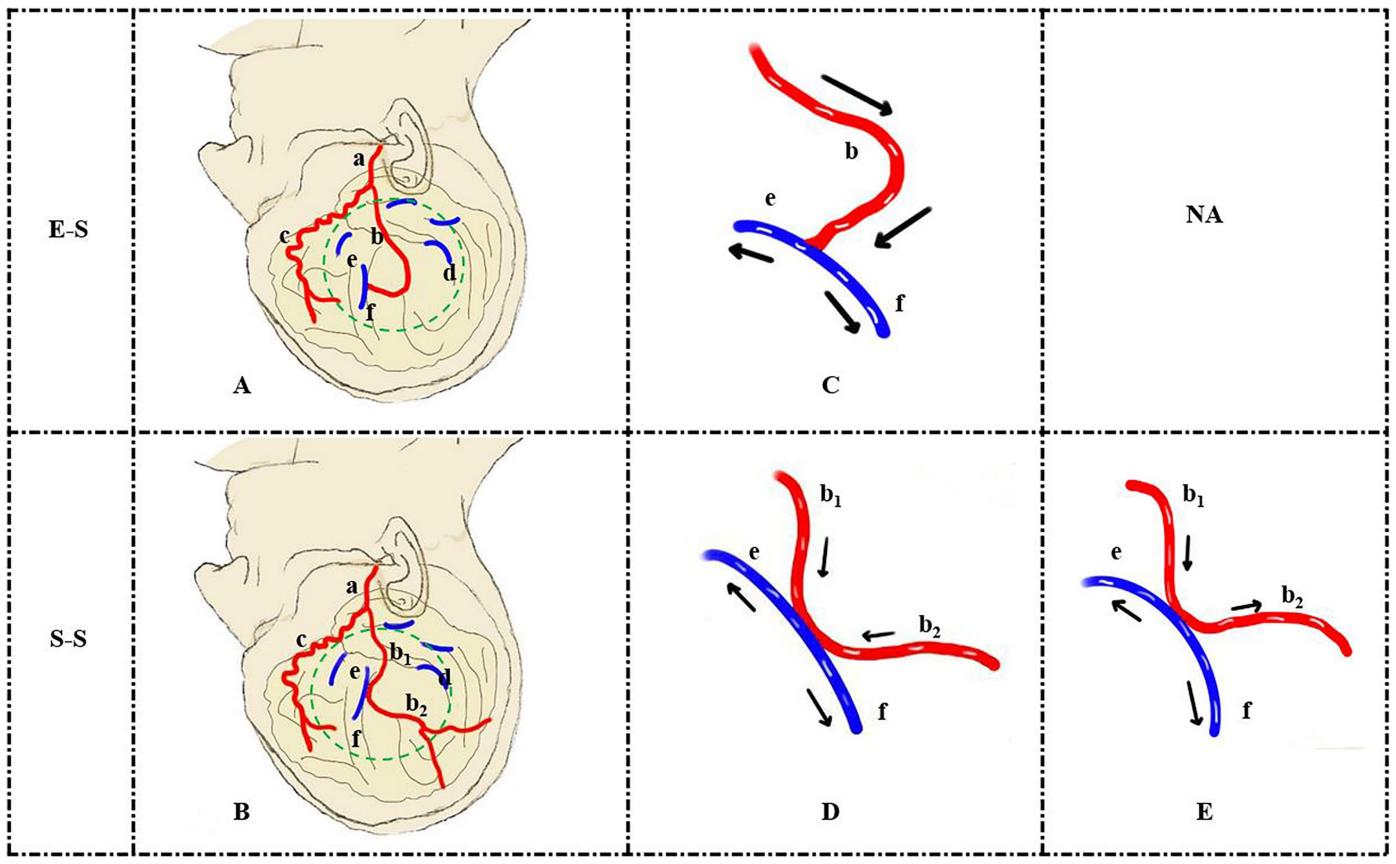
Schematic illustration of E-S **(A,C)** and S-S **(B,D,E)** bypass procedures. Self-flow regulating effects included flow augmentation **(D)** and flow shunting **(E)** effects. (a) The trunk of the STA. (b) The parietal branch of the STA (b1: proximal portion, b2: distal portion). (c) The frontal branch of the STA. (d) The RA of the parietal lobe. (e) RA.ES of the parietal lobe. (f) RA.LS of the parietal lobe. STA, superficial temporal artery; RA, recipient artery; RA.ES, RA entering the Sylvian fissure; RA.LS, RA leaving the Sylvian fissure. The green dotted line represents the craniotomy exposure. NA, not applicable.

The details of the MDU assessment are shown in Figure 3. Before the bypass, the mean velocity values (MVV) for the RA and STA were recorded and expressed as MVV_RA_ and MVV_STA_. Based on the flow direction of the MDU assessments relative to the Sylvian fissure, the RA was categorized as either entering (ES) or leaving (LS) the Sylvian fissure (10). After the bypass, the RA was divided into entering the Sylvian fissure (RA.ES) and leaving the Sylvian fissure (RA.LS) subtypes. In cases undergoing S-S bypass procedures, the DA was anatomically divided into proximal and distal portions relative to the anastomosis site. Hemodynamic effects were classified as either “flow shunting” or “flow augmentation” based on the directional flow patterns observed in these proximal and distal DA portions. When the flow direction of the proximal and distal portions in the DA was the same, DA flow input (DA_input_) was defined as “flow shunting,” expressed as MVV (DA_proximal_) − MVV (DA_distal_). When the flow direction differed, DA_input_ was defined as “flow augmentation,” expressed as MVV (DA_proximal_) + MVV (DA_distal_).

In cases where the E-S technique was used, DA_distal_ was absent, and DA_input_ was equivalent to DA_proximal_. Moreover, the fold change in the MVV of the RA was defined as MVV_RA.ES_/MVV_RA_ and MVV_RA.LS_/MVV_RA_. The flow distribution rate (FDR) of the RA was defined as MVV_RA.ES_/MVV_RA.LS_. The fold change of the DA was defined as MVV (DA_input_)/MVV_STA_ and MVV (DA_proximal_)/MVV_STA_. The MDU results were interpreted by two experienced neurosurgeons.

### 2.5 Perioperative management and complications

Postoperative care adhered to standard protocols, which included maintaining systolic blood pressure at 10% below preoperative levels, rehydrating at 30–50 mL/kg, administering antiplatelet therapy (aspirin, 100 mg/day), preventing seizures, and conducting immediate postoperative CT scans. Repeat CT or additional MRI scans were prescribed to patients with new neurological symptoms. Postoperative complications primarily included cerebral hemorrhage, ischemic stroke, cerebral hyperperfusion syndrome (CHS), and seizures (11). Patients with newly developed discomfort were treated carefully and discharged in stable condition.

### 2.6 Clinical follow-up

The mRS was used to evaluate neurological status at 6 months after surgery, either through clinic visits or telephone interviews. mRS scores of >3, 2–3, and 0–1 were defined as severe disability, moderate disability, and normal, respectively (12). Functional improvement and deterioration were defined as a decrease and an increase in the mRS score compared to preoperative values.

### 2.7 Statistical analysis

Statistical analysis was performed using IBM SPSS 22.0 (IBM Corp.). Pearson’s chi-squared test and Fisher’s exact test were performed for categorical variables. One-way ANOVA was performed for continuous variables with a normal distribution. A rank test was performed on data with a skewed distribution. A *p*-value of <0.05 was considered statistically significant.

## 3 Results

### 3.1 Patient demographics

The baseline characteristics of adult patients with MMD are summarized in Table 1. The cohort consisted of 19 female and 31 male individuals, aged between 19 and 61 years (mean age: 48.9 years). Onset symptoms included hemorrhage (10 cases), headache (14 cases), ischemia (25 cases), and asymptomatic presentation (1 case). The number of cases with hypertension, diabetes, smoking history, hyperthyroidism, and aneurysms were 31 (62%), 16 (32%), 12 (24%), 3 (6%), and 5 (10%), respectively. There were no significant differences between the E-S and S-S groups regarding sex, age, onset symptoms, hypertension, diabetes, smoking history, hyperthyroidism, mRS score, Suzuki stage, moyamoya vessels, operated hemisphere, and aneurysm location (*p* > 0.05).

**Table 1:** The demographics of 50 MMD patients (*n*, %).

Variables	Total (*n* = 50)	E-S (*n* = 27)	S-S (*n* = 23)	*p*
Gender (female, *n*, %)	19 (38.0)	11 (40.7)	8 (34.8)	0.665
Age (year)	48.9 ± 12.6	49.3 ± 11.1	48.6 ± 14.4	0.848
Onset symptom (*n*, %)				0.496
Ischemia	25 (50.0)	17 (63.0)	8 (34.8)	
Hemorrhage	10 (20.0)	2 (7.4)	8 (34.8)	
Headache	14 (28.0)	7 (25.9)	7 (30.4)	
Asymptomatic	1 (2.0)	1 (3.7)	0 (0)	
Hypertension (*n*, %)	31 (62.0)	16 (59.3)	15 (65.2)	0.665
I	2 (6.5)	2 (12.5)	0 (0)	0.365
II	21 (67.7)	10 (62.5)	11 (73.3)	
III	8 (25.8)	4 (25.0)	4 (26.7)	
Diabetes (*n*, %)	16 (32.0)	8 (29.6)	8 (34.8)	0.697
Smoking (*n*, %)	12 (24.0)	7 (25.9)	5 (21.7)	0.842
Hyperthyroidism (*n*, %)	3 (6.0)	1 (3.7)	2 (8.7)	0.459
Aneurysm (*n*, %)	5 (10.0)	3 (11.1)	2 (8.7)	0.578
Peripheral	4 (8.0)	2 (7.4)	2 (8.7)	
Central	1 (2.0)	1 (3.7)	0 (0)	
Operated hemisphere (left, *n*, %)	23 (46.0)	12 (44.4)	11 (47.8)	0.811
Suzuki stage				0.302
Early	4 (8.0)	1 (3.7)	3 (13.1)	
Middle	31 (62.0)	16 (59.3)	15 (65.2)	
Advanced	15 (30.0)	10 (37.0)	5 (21.7)	
Moyamoya vessels				0.785
None	6 (12.0)	4 (14.8)	2 (8.7)	
Sparse	14 (28.0)	7 (25.9)	7 (30.4)	
Dense	30 (60.0)	16 (59.3)	14 (60.9)	
Pre mRS score				0.531
0–1	37 (74.0)	20 (74.1)	17 (73.9)	
2–3	12 (24.0)	7 (25.9)	5 (21.7)	
>3	1 (2.0)	0 (0)	1 (4.3)	

MMD, moyamoya disease; mRS, modified Rankin scale; E-S, end-to-side; S-S, side-to-side.

### 3.2 Comparison of intraoperative hemodynamics

Before the bypass procedure, no significant differences were observed in the MVV and flow direction of the RA between the two groups (*p* > 0.05). After the bypass procedure, the ICG map confirmed immediate bypass patency in 100% of the cases. In patients using S-S technique, 16 of 23 (69.57%) exhibited a flow shunting effect, while 7 out of 23 (30.43%) demonstrated a flow augmentation effect. There was significant difference between the E-S and S-S groups regarding bypass time, anastomosis size relative to the RA, MVV fold changes in the DA_proximal_ and DA_input_, and the MVV of the RA.ES, with the S-S group displaying longer bypass times, larger anastomoses, and higher velocities (*p* < 0.05). No statistical differences were found in the MVV of the DA_proximal_, DA_input_, RA.LS, and FDR (RA.ES/RA.LS) (*p* > 0.05). Moreover, no significant differences were noted in the fold changes of the MVV for the RA.ES and RA.LS (*p* > 0.05) (Table 2).

**Table 2:** Comparison of intraoperative MDU results between E-S and S-S group.

Variables	E-S (*n* = 27)	S-S (*n* = 23)	*p*
Before surgery			
MVV_RA_ (cm/s)	6.18 ± 2.09	8.67 ± 4.53	0.192
RA direction (ES, *n*, %)	15 (55.6%)	9 (39.1%)	0.247
Anastomosis size/RA	1.67 (1.31, 1.83)	3.18 (2.91, 3.26)	<0.001**
After surgery			
Bypass time (min)	16.6 ± 4.5	27.6 ± 3.9	<0.001**
DA_input_ (cm/s)	49.46 ± 25.31	62.22 ± 17.35	0.169
DA_proximal_ (cm/s)	49.46 ± 25.31	65.67 ± 19.07	0.087
RA.ES (cm/s)	30.04 ± 19.83	59.78 ± 25.02	<0.001**
RA.LS (cm/s)	25.25 ± 16.25	31.56 ± 18.66	0.335
FDR (RA.ES/RA.LS)	1.00 (0.65, 2.01)	1.62 (1.17, 3.41)	0.100
Folds change			
MVV_RA.ES_	4.67 (3.40, 7.06)	7.17 (4.33, 14.21)	0.134
MVV_RA.LS_	3.38 (2.39, 7.31)	2.86 (1.89, 7.60)	0.492
MVV_DA input_	4.41 ± 2.25	7.29 ± 4.53	0.015*
MVV_DA proximal_	4.41 ± 2.26	6.78 ± 4.00	0.031*
Flow shunting of STA (*n*, %)	0	16 (69.57%)	<0.001**

FDR, flow distribution ration; MDU, microvascular Doppler ultrasonography; RA, recipient artery; MVV, mean velocity value; DA, donor artery; RA.ES, recipient artery entering Sylvian; RA.LS, recipient artery leaving Sylvian; DA_proximal_, proximal portion of donor artery; E-S, end-to-side; S-S, side-to-side. “*” was *p* < 0.05, “**” was *p* < 0.01. Bold represented the *p* value < 0.05.

### 3.3 Comparison of postoperative complications

Of 50 cases (24%), 12 developed postoperative complications, including new infarction in 5 (10%) cases, hemorrhage in 5 (10%) cases, CHS in 7 (14%) cases, and seizure in 1 (2%) case. There was no statistical difference between the E-S and S-S groups regarding the incidence of postoperative infarction, hemorrhage, CHS, seizure attack, and clinical prognosis at discharge (*p* > 0.05). Of the seven cases with CHS, the symptom distribution varied between the groups: three cases (13%) in the S-S group experienced hemorrhage, while two cases (7.4%) in the E-S group (*p* = 0.508) had hemorrhage (Table 3).

**Table 3:** Comparison of postoperative complication and clinical follow-up between E-S and S-S group (*n*, %).

Variables	Total (*n* = 50)	E-S (*n* = 27)	S-S (*n* = 23)	*p*
Complication	12 (24.0)	6 (22.2)	6 (26.1)	0.750
Infarction	5 (10.0)	2 (7.4)	3 (13.0)	0.651
Ipsilateral	3 (6.0)	1 (3.7)	2 (8.7)	
Contralateral	2 (4.0)	1 (3.7)	1 (4.3)	
ACA/MCA	5 (10.0)	2 (7.4)	3 (13.0)	
PCA	0 (0)	0 (0)	0 (0)	
Hemorrhage	5 (10.0)	2 (7.4)	3 (13.0)	0.508
CHS	7 (14.0)	4 (14.8)	3 (13.0)	0.857
Seizure	1 (2.0)	1 (3.7)	0 (0)	0.351
Prognosis				0.210
Improvement	22 (44.0)	9 (33.3)	13 (56.5)	
Deterioration	9 (18.0)	5 (18.5)	4 (17.4)	
No change	19 (38.0)	13 (48.2)	6 (26.1)	
Matsushima grade at 6-months follow-up				0.571
A	20 (40.0)	9 (33.3)	11 (47.8)	
B	23 (46.0)	14 (51.9)	9 (39.1)	
C	7 (14.0)	4 (14.8)	3 (13.0)	
Bypass patency, follow-up	43 (86.0)	23 (85.2)	20 (87.0)	0.857
mRS score, follow-up				0.413
0–1	36 (72.0)	21 (77.8)	15 (65.2)	
2–3	13 (26.0)	6 (22.2)	7 (30.4)	
>3	1 (2.0)	0 (0)	1 (4.4)	

ACA, anterior cerebral artery; MCA, middle cerebral artery; PCA, posterior cerebral artery; CHS, cerebral hyperperfusion syndrome; E-S, end-to-side; S-S, side-to-side.

### 3.4 Comparison of hemispheric parameters

Before surgery, there was no significant difference between the E-S and S-S groups regarding the following measurements: volume of CBF <30%, volume of *T*_max_ >6 s, volume of *T*_max_ >10s, volume of penumbra, hypoperfusion index, collateral vessel density, and mismatch ratio (*p* > 0.05). After achieving BP control post-bypass, significant differences were found between the groups in terms of collateral vessel density, mismatch ratio, volume of CBF <30%, *T*_max_ >6 s, *T*_max_ >10 s, hypoperfusion index, and the volume of penumbra (*p* > 0.05). Moreover, there were no significant differences between the E-S group and S-S group in the percentage of the infarcted core (CBF <30%), hypoperfusion (*T*_max_ >6 s), and changes in the penumbra volume (Table 4).

**Table 4:** Comparison of postoperative hemispheric perfusion between E-S and S-S group.

Variables	E-S (*n* = 27)	S-S (*n* = 23)	*p*
Before surgery			
Collateral vessel density	0.008 (0.002, 0.019)	0.014 (0.005, 0.016)	0.359
CBF <30% (mL)	2.50 (1.01, 38.85)	19.25 (8.72, 47.03)	0.334
*T*_max_ >6 s (mL)	84.30 (14.5, 168.40)	100.81 (73.08, 182.08)	0.443
*T*_max_ >10s (mL)	0 (0, 4.10)	1.3 (0, 13.3)	0.711
Hypoperfusion index	0 (0, 0.02)	0.015 (0, 0.073)	0.604
Penumbra (mL)	82.00 (14.5, 129.7)	80.45 (48.38, 134.55)	0.711
Mismatch ratio	4.6 (1.0, 9.3)	4.40 (2.28, 10.41)	0.786
After surgery			
Collateral vessel density	0.087 (0, 0.03)	0.018 (0.007, 0.046)	0.187
CBF <30% (mL)	13.40 (0, 46.60)	16.95 (9.25, 39.03)	0.570
ΔCBF <30% (mL)	0 (−0.053, 0.210)	−0.034 (−0.411, 0.757)	0.711
Percentage of ΔCBF <30% (%)	0 (−52.6, 20.1)	−34.4 (−41.2, 75.8)	0.834
*T*_max_ >6 s (mL)	133.40 (7.10, 182.20)	83.8 (47.1, 103.2)	0.505
Δ*T*_max_ >6 s (mL)	0 (−0.61, 0.63)	−0.31 (−0.46, −0.14)	0.359
Percentage of Δ*T*_max_ >6 s (%)	0 (−60.8, 63.1)	−31.1 (−45.7, −14.5)	0.410
*T*_max_ >10s (mL)	0 (0, 2.60)	1.4 (0, 8.3)	0.571
Hypoperfusion index	0 (0, 0.24)	0.023 (0, 0.081)	0.414
Penumbra (mL)	72.0 (1.10, 135.60)	59.6 (33.32, 70.68)	0.537
ΔPenumbra (mL)	0 (−0.92, 0.16)	−0.46 (−0.60, 0.11)	0.604
Percentage of Δpenumbra (%)	0 (−92.1, 16.3)	−45.6 (−60.4, 10.3)	0.736
Mismatch ratio	3.10 (0, 6.50)	3.65 (1.98, 5.41)	0.639

CBF, cerebral blood flow; *T*_max_, time to max; percentage of Δ = [volume (_post_) − volume (_pre_)]/volume (_pre_) * 100%; E-S, end-to-side; S-S, side-to-side.

### 3.5 Comparison of clinical follow-up data

**Figure 4:**
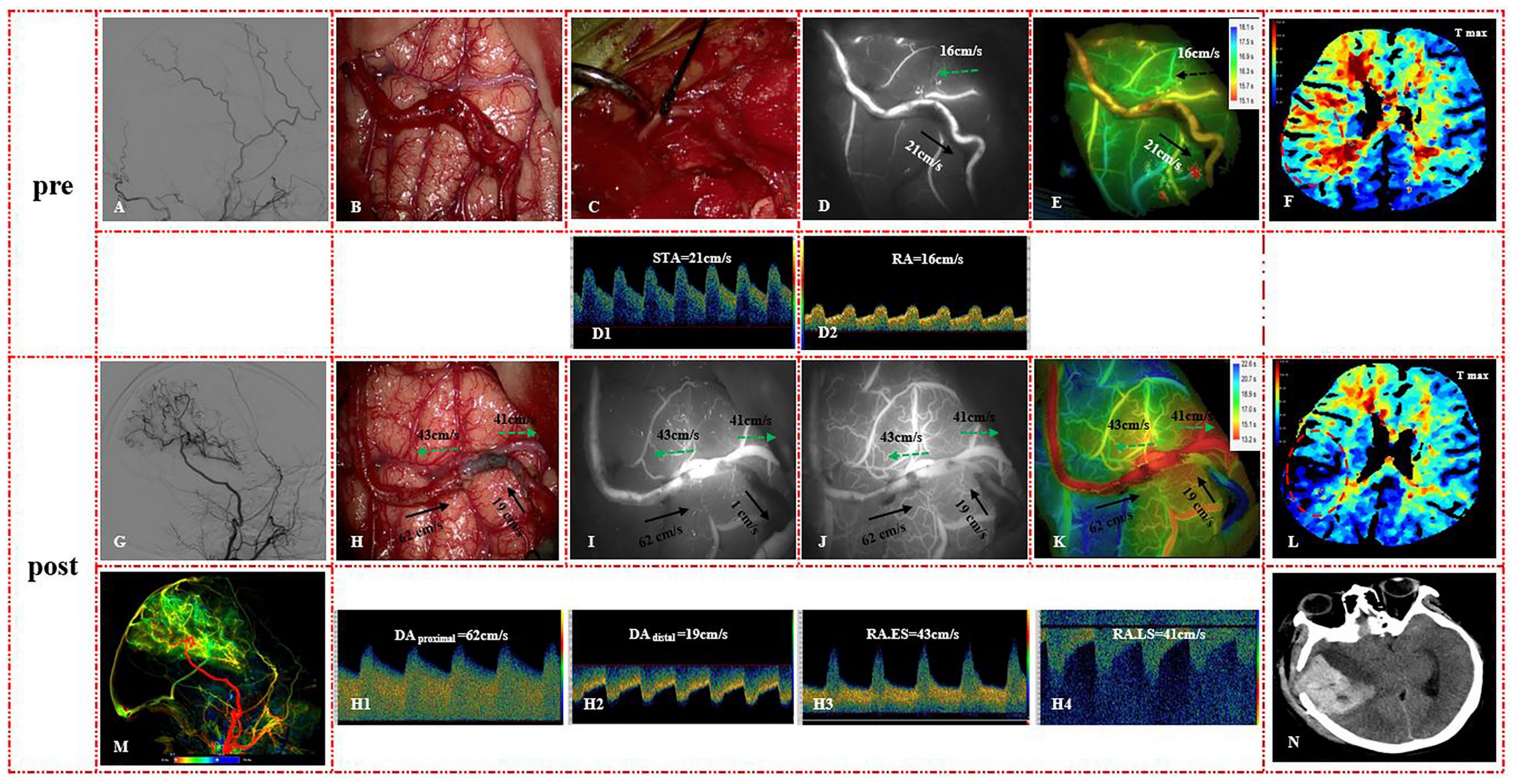
**(A)** Lateral view of the ECA on preoperative DSA. **(B)** Difficulty in anastomosis due to the distance between the STA and RA. **(C)** Ligation and transection of the frontal branch of the STA. **(D–F)** ICG angiography, Flow 800, and CT perfusion images before the bypass. **(D1,D2)** MVV of the STA and RA before the bypass. **(G,M)** Postoperative DSA demonstrated bypass patency. **(H)** MDU results after the bypass. The MVV of DA_proximal_ **(H1)**, DA_distal_ **(H2)**, RA.ES **(H3)**, and RA.LS **(H4)** was 62 cm/s, 19 cm/s, 43 cm/s, and 41 cm/s, respectively. **(I)** The first ICG angiography demonstrated that the DA_distal_ developed slowly, with a slow outflow velocity of 1 cm/s. **(J)** The second ICG angiography showed that DA_distal_ began to develop in reverse, with an inflow velocity of 19 cm/s. **(K)** Flow 800 images of the second ICG examination. **(L)** Postoperative CTP showed perfusion improvement (red circle). **(N)** CT scan at 3 days after the bypass demonstrated a new brain hemorrhage.

At clinical follow-up, no patient experienced a new ischemic or hemorrhagic stroke since discharge. Bypass patency showed no significant differences between the groups, with 23/27 cases (85.2%) in the E-S group and 20/23 cases (87%) in the S-S group (*p* = 0.857). Matsushima grade A was achieved in 9 out of 27 patients (33.3%) in the E-S group and 11 out of 23 patients (47.8%) in the S-S group, showing no statistical differences between the groups (*p* = 0.571). In addition, the mRS scores did not differ significantly between the groups (*p* > 0.05) (Figure 4).

### 3.6 Illustrated case

A 48-year-old female patient presented with left limb numbness and was diagnosed with MMD. She was subsequently treated with an S-S STA-MCA bypass. Intraoperative images revealed challenges in performing the anastomosis due to the significant distance between the STA and RA. To alleviate vascular tension, the frontal branch of the STA was ligated and transected. MDU revealed MVVs of 21 cm/s and 16 cm/s for the STA and RA before bypass. After bypass, ICG angiography confirmed bypass patency. The first ICG angiography demonstrated that DA_distal_ developed slowly with an outflow velocity of 1 cm/s (flow shunting effect). After 20 min, the second ICG angiography showed that DA_distal_ began to develop in reverse with an inflow velocity of 19 cm/s (flow augmentation effect). MDU revealed an MVV of 62 cm/s, 19 cm/s, 43 cm/s, and 41 cm/s in the DA_proximal_, DA_distal_, RA.ES, and RA.LS, respectively. Postoperative DSA and CTP revealed a well-developed bypass with improved perfusion in the right parietal lobe. However, a CT scan on postoperative day 3 revealed a new brain hemorrhage, necessitating urgent hematoma evacuation (Figure 4).

## 4 Discussion

The S-S technique used in the EC-IC bypass procedure has been previously described (3). However, its safety, efficacy, intraoperative hemodynamics, and hemispheric perfusion under BP control remain unclear. The advantage of S-S anastomosis may contribute to the STA’s self-regulation phenomenon, potentially reducing the incidence and symptom duration of postoperative CHS events (4). In this study, there was no significant difference between the E-S and S-S groups in terms of the incidence of postoperative new infarctions, cerebral hemorrhage, CHS, and epilepsy. The incidence of CHS in the E-S group (14.8%) was nearly equal to that in the S-S group (13%). However, among the five cases with new hemorrhage after the bypass procedure, the S-S group had a slightly higher incidence (3/23, 13%) compared to the E-S group (2/27, 7.4%). We hypothesized that the STA plays two different roles in cases where the S-S technique is used, functioning either as flow shunting or flow augmentation, depending on the flow direction of DA_distal_.

**Figure 5:**
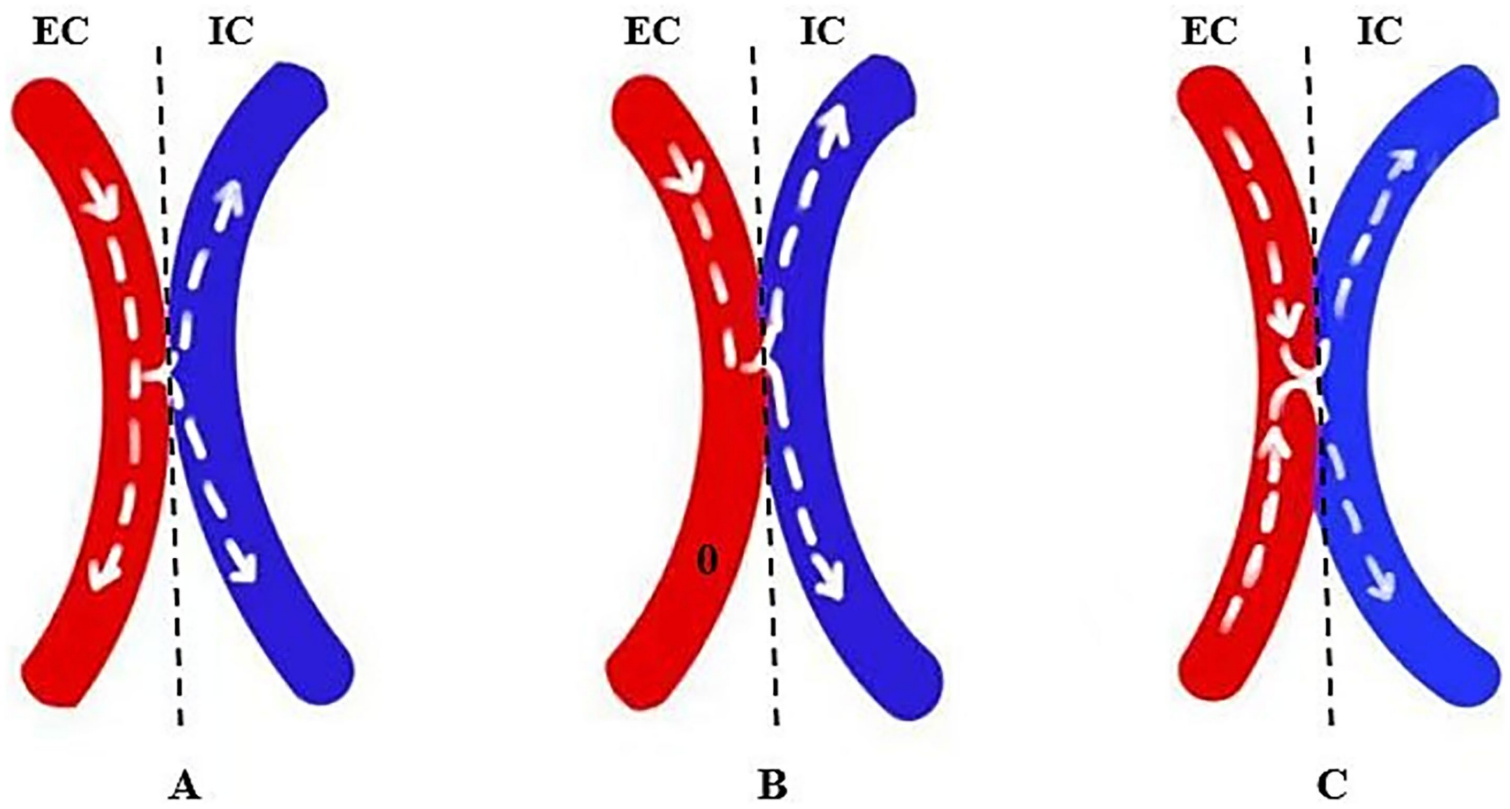
Schematic diagram of flow distribution in the DA. **(A)** When the anastomotic orifice is too small, blood flow is shunted to the distal portion of the DA. **(B)** As the anastomotic orifice gradually enlarges, the shunting effect diminishes. **(C)** When the anastomotic orifice becomes oversized, the distal portion of the DA supplies blood intracranially.

To the best of our knowledge, this is the first report characterizing such distinct flow-dependent functional duality of the STA in cerebral revascularization, as no similar reports have been published previously. In patients undergoing the S-S procedure, 16 out of 23 (69.57%) exhibited flow shunting, while 7 out of 23 (30.43%) exhibited flow augmentation. We hypothesize that these differences may be associated with the size of the anastomosis and the pressure gradient. According to Poiseuille’s law (13), theoretical blood flow (*Q*_theoretical_) is proportional to the EC-IC pressure gradient (ΔP) and the fourth power of the anastomosis radius (*r*^4^). If the S-S case with ΔP or anastomosis radius is too large, *Q*_theoretical_ may exceed the blood supply of DA_proximal_, requiring DA_distal_ to supply blood, which results in a flow augmentation effect. Conversely, if *Q*_theoretical_ is less than the flow from the DA_proximal_, then the DA_distal_ has a flow-shunting role. Furthermore, we speculated that the anastomosis size, representing a controllable factor, may play an important role in the “flow shunting” or “flow augmentation” effects. If the anastomosis is too small, it may restrict the inflow of blood from the DA into the RA (*Q*_theoretical_ < STA_proximal_), thereby shunting the DA flow toward the scalp, resulting in a “flow shunting” effect. As the size of the anastomosis increases, *Q*_theoretical_ gradually approaches the STA_proximal_, leading to a progressive flow reduction in the DA_distal_. However, it remains unclear whether a state of zero flow in the DA_distal_ can be achieved. Conversely, if the anastomosis is excessively large (*Q*_theoretical_ > STA _proximal_), the DA_distal_ may be required to contribute to blood supply, resulting in a “flow augmentation” effect (Figure 5). Therefore, we speculate that the anastomosis size should not be too large in the S-S procedure, with an optimal longitudinal/transverse diameter ratio between 1.0 and 2.0. An oversized anastomosis (>2.5) may lead to excessive flow augmentation, potentially exceeding the hemodynamic tolerance of the vascular bed in the RA. Subsequent fluctuations in blood pressure (such as those occurring during bowel movements, severe coughing, or fever) could then cause vascular overload and hemorrhage. This hypothesis is novel and requires further investigation. However, it remains unclear how to improve the bypass technique to achieve a random control of flow shunting or slow augmentation.

Previous studies have primarily focused on intraoperative ICG color mapping, with fewer reports leveraging intraoperative ultrasonography (14, 15). In our previous report, intraoperative TCD was used to evaluate hemodynamics, identifying that MVV fold changes in RA.ES are a risk factor for CHS (10). In the current series, the MVV of RA.ES and MVV fold changes in the DA (DA_proximal_ and DA_input_) in the S-S group were significantly higher than those in the E-S group (*p* < 0.05). Other intraoperative variables showed no significant difference between the patients undergoing the E-S and S-S procedures. Although there were no significant differences, the MVV and fold changes in the MVV of the RA.ES segment were higher than those of the RA.LS segment. Accordingly, we speculate the following: (1) a self-flow regulation phenomenon exists both in the DA and RA, exhibiting dynamic adaptability; (2) the self-flow regulation ability of donor vessels is stronger in the S-S bypass than in the E-S bypass; (3) the self-flow regulation of the RA favors RA.ES over RA.LS; and (4) the RA has a certain capacity to tolerate flow input and a self-regulating ability to achieve an optimal distribution, which does not increase indefinitely with increasing blood flow from the donor vessel. These speculations are novel and need further validation.

According to previous reports, EC-IC bypass can increase blood flow to the ipsilateral hemisphere (16–18). However, no prior studies have addressed hemispheric hypoperfusion during active systolic BP control aimed at preventing CHS. After the EC-IC bypass procedure, there was no significant difference between the E-S and S-S groups in the volume of the infarct core, hypoperfusion, penumbra, and collateral vessel density (*p* > 0.05). However, compared to the E-S group, the S-S group showed no significant advantage in reducing volume changes in the infarct core, hypoperfusion regions, or penumbra after the surgery (*p* > 0.05). This suggests that the S-S group had no superiority over the E-S group in reducing infarction and hypoperfusion volumes—an observation not previously reported.

Moreover, EC-IC bypass is known to facilitate neovascularization and improve clinical prognosis (19, 20). At the latest follow-up, no patient experienced recurrent ischemia or hemorrhage events. Compared to the E-S bypass, the S-S bypass appeared to be more favorable in achieving Matsushima grade A and bypass patency. However, there was no significant difference in Matsushima grade A, bypass patency, and the short-term mRS score between the two groups. These encouraging results suggest that the S-S bypass is not inferior to the E-S bypass, although further studies are needed to assess potential differences in long-term outcomes.

## 5 Limitations

This study has some limitations. Firstly, the sample size was limited, increasing the risk of type I statistical errors. Therefore, there is a need to enroll more patients in future studies. Secondly, this study relied on the MVV derived from the MDU technique for hemodynamic comparisons, rather than direct cut flow measurements, which are more intuitive. Future studies should incorporate ultrasound flow probes for more precise monitoring (21). Thirdly, CT perfusion provides semiquantitative blood flow measurements, which are not as accurate as single-photon emission computed tomography (SPECT) or positron emission tomography (PET) (22). Finally, the follow-up period was relatively short and did not reveal significant differences. Therefore, a longer follow-up period is needed.

## 6 Conclusion

The application of the S-S technique exhibited distinct intraoperative self-flow regulation capacities. However, this research showed that the S-S bypass yielded no superiority in clinical and radiological outcomes compared to the standard E-S bypass. Routine intraoperative hemodynamic assessments provided deeper insights into the physiological interactions between the bypass and recipient vessels. These findings emphasize the importance of tailoring EC-IC bypass options based on each patient’s specific vascular anatomy and hemodynamic factors.

## Data Availability

The raw data supporting the conclusions of this article will be made available by the authors, without undue reservation.
